# The Use of Infliximab (Remicade®) for the Treatment of Rheumatic Diseases at a Tertiary Center in Lebanon: A 17-Year Retrospective Chart Review

**DOI:** 10.31138/mjr.31.4.400

**Published:** 2020-12-22

**Authors:** Vicky Nahra, Georges El Hasbani, Monique Chaaya, Imad Uthman

**Affiliations:** 1 Division of Rheumatology, Department of Internal Medicine, American University of Beirut, Medical Center, Beirut, Lebanon; 2 Department of Epidemiology and Population Health, Faculty of Health Sciences, American University of Beirut, Medical Center, Beirut, Lebanon

**Keywords:** Biologics, infliximab, indications, efficacy, safety profile

## Abstract

**Objectives:**

Infliximab (Remicade®) was the first tumour necrosis factor-α (TNF) inhibitor to receive its initial marketing approval from the US Food and Drug Administration (FDA) for the treatment of Crohn’s disease. Following that, infliximab became approved for several immune-mediated inflammatory diseases. No evidence exists in the Middle East and North Africa region on the experience with infliximab use over an extended period in terms of efficacy and safety.

**Methods:**

The Rheumatology division at the American University of Beirut Medical Centre (AUBMC), one of the largest tertiary centres in the Middle East and North Africa region, has been using infliximab infusions for the treatment of certain rheumatic diseases for around two decades. By reviewing retrospectively medical charts at AUBMC, we investigate indications, safety and efficacy, rate of withdrawals, rate of switching to another biologic, and financial coverage of the drug to present data for practitioners and patients in the region considering infliximab for treatment of immune-mediated inflammatory diseases.

**Results:**

A total of 198 patients were identified in the past 17 years to have taken infliximab. The largest proportion of treated patients had RA. Fourteen percent of the total cohort experienced serious adverse events, with 96.4% of those events being mild hypersensitivity reactions. Five patients withdrew the medication because of infectious complications, 4 of which were cases of tuberculosis reactivation. Despite that, around half of the patients were switched to another biologic agent such anti-TNF-α, anti-CD20, and anti-IL-6 due to partial response, and less than half were receiving add-on disease-modifying anti-rheumatic drugs (DMARDs) such as methotrexate, 70% of patients who used infliximab only or were switched achieved complete remission at their last hospital information. Around 98% of infliximab users were financially covered.

**Conclusion:**

According to our experience, infliximab has made remission and prevention of long-term disability realistic goals of therapy in the Middle East region.

## INTRODUCTION

Infliximab is a chimeric monoclonal antibody that targets the cytokine TNF alpha. It was first approved in 1998 for the treatment of Crohn's disease (CD).^1^ In November 1999, the U.S. Food and Drug Administration (FDA) approved the use of infliximab for Rheumatoid Arthritis (RA) with methotrexate and elaborated this indication in December 2000.^2^ Currently, the FDA indicates the use of infliximab for RA, Ankylosing Spondylitis (AS), Psoriatic Arthritis (PsA), Crohn’s Disease (CD), and Ulcerative Colitis (UC).

The use of infliximab was first introduced by the Rheumatology unit at The American University of Beirut Medical Centre (AUBMC), a tertiary care centre and teaching hospital, and one of the largest medical centres in the region, in October 2000.^3^ Although two previous studies have reported the use of biological treatments in chronic inflammatory diseases in Lebanon,^3,4^ none of these investigated the use of a single biological agent with its different indications over a period of 17 years.

The use of infliximab is not limited to the previously mentioned indications. In a former study at AUBMC,^4^ the use of infliximab was remarkable in chronic refractory rheumatic diseases other than the FDA approved ones. These included: Behçet’s disease, Behçet uveitis, Sjögren's syndrome, polymyositis, Sarcoidosis, Takayasu disease, Cogan's disease, Stills disease, Mixed Connective Tissue Diseases (MCTDs), deep cutaneous vasculitis, and subacute cutaneous lupus erythematosus.

Because sufficient data in the Middle East and the Arab region is still lacking, the aim of this study is to describe all the chronic inflammatory conditions in which infliximab was prescribed at a tertiary centre in Lebanon over a period of two decades, and to report the rate of remissions, relapses, withdrawals, and side effects.

## PATIENTS AND METHODS

### Research design

This study is a retrospective electronic medical chart review. The patient population included those who have received infliximab infusions for different indications at the rheumatology unit at AUBMC, from October 2000 (when infliximab first became available at our centre) until May 2017.

Data concerning infliximab use indications, safety and efficacy, adverse events, rates of withdrawals from the treatment, switching to other biological treatments, and the drug’s financial coverage, were collected. Approval for this study was granted by the Institutional Review Board at AUBMC.

A data collection sheet was prepared and approved, which included all needed parameters in question for the review, and was filled for each patient upon performing an electronic chart review by the medical research fellow, with all data available in the patient’s charts including gender, age, history of present illness, past medical history, social history, family history, diagnosis, treatment, duration of treatment, and insurance status.

### Statistical analysis

Data obtained was entered and analysed using SPSS 17.0, using descriptive statistics.

## RESULTS

A total of 207 patients treated with infliximab for different rheumatologic diseases were identified for inclusion into the study. Seven patients were excluded due to lack of follow-up data. The total number of included patients was 198.

The age of patients ranged from 18 to 88 years, with a mean age of 49 years (± 2.5). Females constituted 54.5% of the total patients. More than one third were smokers (39.1%), and 11.2% were current alcohol drinkers.

### Indications

The largest proportion of patients who were on infliximab had RA (33%), either seronegative or seropositive (not differentiated) followed by AS (20.8%) and PsA (11.2%). Patients with Behçet’s disease and Autoimmune Hearing Loss (AIHL), comprised respectively 4.6% and 4.1 % of the selected patients. Sarcoidosis, Inflammatory Bowel Disease (IBD)-related arthritis, Uveitis, and Takayasu’s arteritis, each constituted 3.6 % of the patient population. The last group of infliximab indications was pooled into one category labelled as “others” due to the small number in each disease category, and it constituted 12.2% or a total of 24 patients. It consists of: 4 patients with undifferentiated spondylarthritis (SpA), 2 patients with MCTD, 2 patients with Sjogren’s Syndrome (SS), 2 patients with Amyloidosis, 1 patient each with the following: Sicca Syndrome, Systemic Lupus Erythematosus (SLE), Pyoderma Gangrenosum (PG), Cutaneous Vasculitis, Still’s disease, Giant Cell Arteritis (GCA), Iridocyclitis, Polyarthritis, Myositis, Sacroiliitis, and Common Varied Immune Deficiency (CVID). **Table 1** summarises the data on the indications of infliximab use.

**Table 1.** The percentage and absolute number of patients with each specific indication for infliximab infusion.

**Indications**	**Frequency (n)**	**Percentage of patients (%)**
**RA**	65	33
**AS**	41	20.8
**PsA**	22	11.2
**Behçet’s disease**	9	4.6
**AIHL**	8	4.1
**Sarcoidosis**	7	3.6
**IBD arthritis**	7	3.6
**Uveitis**	7	3.6
**Takayasu’s arteritis**	7	3.6
**Others**	24	12.2

One patient in our cohort had no diagnosis, and was thus excluded from this table.

Acronyms: RA: Rheumatoid Arthritis; AS: Ankylosing Spondylitis; PsA: Psoriatic Arthritis; AIHL: Autoimmune hearing loss; IBD: Inflammatory Bowel Disease.

### Safety and Efficacy

Regarding infliximab infusions, the first one was given on October 24, 2000. The dates recorded in this study ranged from October 24, 2000 till April 21, 2017. The number of infusions ranged from 1 to 80. The mean was 13.32 ± 1.95 infusions, with 9.3% of patients taking 4 injections.

Infusion adverse events (AE) were reported by the physicians in the charts. The majority (85.8%) of patients did not experience any adverse event that was reported in the electronic medical records (EMR). Twenty-eight patients (14.2%) developed an AE. Seven had RA, 8 had AS, 3 had PsA, 2 had AIHL, 2 had sarcoidosis, 3 had IBD arthritis, 1 had Takayasu’s arteritis, and 2 had others. Of the patients who developed an AE, 96.4% of the cases were allergies to the medication ranging from itching, rash, to fever and hypotension. One patient with RA developed idiopathic pulmonary fibrosis diagnosed by chest CT scan 3 months after starting the treatment. Infliximab was given according to the standard dosing regimen and frequency.

The dose depends on the indication of use. In our study, the frequency of infusion of the drug ranged from every 2 months to every 6 months or yearly depending on the patient’s symptoms. The most frequent was every 2 months at 48.6% of the patients, followed by a variable dosing (as per patient’s symptoms) at 40.5%, and finally every 3 months at 10.9%. The dose of Infliximab ranged from 100 mg to 600 mg, with a mean of 316.38 mg ± 14.74 mg. Although the dose of 100 mg is usually not given, 1 patient with RA was given a dose of 100 mg. The most frequent dose was 300 mg with 32.8% of patients, followed by 200 mg with 28.8%, then 400 mg with 27.7%, then 500 mg with 9% and finally 100 mg and 600 mg with 1.1% and 0.6%, respectively.

The patients’ rheumatologic disease and drug statuses were assessed by reviewing their last follow up appointment or last hospital admission details on EMR after taking infliximab, if available. The categories ranged from remission to active disease. Seventy percent of patients were in remission, 15.2% had moderately active disease, 9.4% had mildly active disease, 1.4% had highly active disease, 2.9% were deceased and 1.4% were lost to follow up. Of those in remission, 17% achieved it in less than 6 months, 5.7% between 6 and 12 months, and 77.3% in more than 1 year. Infliximab status data showed that 90% of the patients were off the drug and only 10% were still taking the infusion.

### Withdrawal

Reasons for withdrawal of the medication varied: 64% due to loss of efficacy, 10% due to allergic reactions, 5% due to infections, 2% due to malignancy, and 19% grouped as other causes, representing 18 patients; 12 patients who had controlled disease, one who became pregnant, one who developed pulmonary fibrosis, and four were unknown.

The withdrawn patients who developed infections on infliximab were only 5 cases (5%), 4 (80%) of which developed tuberculosis reactivation and 1 (20%) had recurrent upper respiratory tract infections. The patient who developed tuberculosis infection had a negative tuberculin skin test (TST) prior to starting infliximab. None of these infections was life threatening. The patients who were diagnosed with malignancy after infliximab (2%) were only two cases; one had a non-Hodgkin’s lymphoma and the other had bladder papillary urothelial carcinoma.

### Switching

Around half the patients (48.7%) were switched to another biologic agent. Of those who were switched, the medication they received after infliximab are presented in **Table 2**. The most common medications received after infliximab were anti-TNF (46.8 %) and anti-CD20 (36.4 %). Almost all improved after the switch (95.8 %).

**Table 2.** Percentage of patients receiving each drug class after being switched to another biologic agent following infliximab withdrawal.

**Drug Class**	**Percentage of patients (%)**
**Anti-TNF**	46.8
**Anti-CD20**	36.4
**Anti-IL6**	13.0
**Abatacept**	2.6
**Others**	1.3

Others include: Undifferentiated spondylarthritis (SpA), Mixed connective tissue disease (MCTD), Sjogren’s Syndrome (SS), Amyloidosis, Polyarthritis, Relapsing Polychondritis, Sicca Syndrome, Systemic Lupus Erythematosus (SLE), Pyoderma Gangrenosum (PG), Cutaneous Vasculitis, Still’s disease, Giant Cell Arteritis (GCA), Scleroderma, Iridocyclitis, Myositis, and Common Varied Immune Deficiency (CVID).

Acronyms: TNF: Tumour necrosis factor; IL: Interleukin.

### Add-ons

Less than half the patients were receiving disease-modifying anti-rheumatic drugs (DMARDs) with infliximab (41.7%). **Table 3** provides data on the DMARDs used by our patients. The most used add-on was methotrexate.

**Table 3.** Percentage of patients on different Disease modifying anti-rheumatic drugs (DMARDs).

**DMARD**	**Percentage of patients (%)**
**Methotrexate**	62.7
**Antimalarials**	4.5
**Leflunomide**	9
**Azathioprine**	6
**Mycophenolate Mofetil**	6
**Colchicine**	11.9

Besides DMARDs, 18.5% of those on infliximab were receiving steroids. The latter were on different doses of Prednisone, ranging from 1.25 to 40 mg, with a mean dose of 14 mg +/– 5.13 mg (95% CI).

### Financial coverage

Almost all (97.5%) had insurance: 62.7% of patients were covered by private insurance, 24.1% by the National Social Security Fund (NSSF), 10% by the Ministry of Public Health (MOPH) and 0.6% by the Army. Only 2.5% were self-payers. The dose and frequency of infliximab infusions were the same in self-payers and insurance covered patients.

## DISCUSSION

In our retrospective electronic medical chart review, 65% of the total patients had at least one disease for which infliximab use is FDA approved. Infliximab showed good efficacy on the articular manifestations of IBD in the literature,^5^ which explains that 3.6% of all patients had IBD arthritis and were using infliximab as a potential mean for remission.

We observed that a low proportion of study population experienced a drug adverse event of which the largest proportion was hypersensitivity reactions. The long-term use of infliximab has been linked with infectious and non-infectious pulmonary events. Tuberculosis represents one of the various infectious entities. An increased incidence of pulmonary mycobacterial infections and atypical mycobacterial infections has been reported.^6^ In addition, Winthrop and colleagues^7^ reported 239 cases of nontuberculous mycobacteria infections in the setting of anti–TNF-α use of which 73 cases were associated with infliximab use. Four cases in the present study had tuberculosis reactivation following drug use. Besides the infectious pulmonary complications, several non-infectious conditions such as ILD have been reported in the literature. The incidence of ILD following anti-TNF-α use in RA patients was assessed by Curtis and colleagues.^8^ When the sensitive definition of ILD was used, unadjusted incidence rates (95% confidence interval, or CI) reached 12.2 (5.6–23.2) per 1000 person-years in the infliximab group. Despite such high incidence, only 1 patient withdrew infliximab because of ILD event in our study. Expanding our cohort by the inclusion of other autoimmune and autoinflammatory diseases using infliximab would have given better conclusions about the incidence of ILD as a side-effect among infliximab users.

About 30–40% of patients who use anti-TNF-α drugs fail to achieve the clinical target or to maintain an initial good response over time, or experience adverse events leading to treatment withdrawal.^9^ However, our data showed that 70% of patients were in remission at their last hospital information. Moreover, 17% of patients in remission achieved it in less than 6 months. Because the response to anti-TNF-α can be partial, around half of our patients were switched to different disease-modifying anti-rheumatic drugs (DMARDs). According to the literature, some partial responders may benefit from switching to a different biologic DMARD with the same or different mechanism of action. The practice of switching between different anti-TNF-α drugs (cycling strategy) has become widespread in the 2000s as a result of limited alternative options.^10^ In Cohen and colleagues cohort study,^11^ 18 out of 24 RA patients who were switched from infliximab to another anti-TNF-α drug reported significant decrease of disease activity score (DAS) in 28 joints measurements and CRP values. Similarly, a significant reduction from baseline in DAS 28-C Reactive Protein (DAS28-CRP), Simplified Disease Activity Index (SDAI), and Clinical Disease Activity Index (CDAI) was observed at different time points in RA patients who were switched from infliximab with methotrexate to tocilizumab (Anti-IL-6 drug) with or without methotrexate in Wakabayashi et al. study.^12^ In the SWITCH-RA prospective study, 604 patients received rituximab and 507 patients received an alternative anti-TNF-α drug after initial withdrawal of an anti-TNF-α drug. The study concluded that switching to rituximab is associated with significantly improved clinical effectiveness compared with switching to a second TNF-α by assessing the change in DAS28 excluding patient's global health component (DAS28-3)-erythrocyte sedimentation rate (ESR) over 6 months.^13^ The promising results of such studies explain switching our patients to another anti-TNF-α, anti-CD20, and anti-IL-6.

Since the chronic use of infliximab has been associated with loss of response and formation of antibodies to DMARDs,^14^ many DMARDs have been used in combination to maintain remission. This could explain in part that a large proportion of our patients were on other DMARD while using infliximab. The systemic review by Costa and colleagues^15^ concluded that the combination of infliximab and methotrexate is more effective in treating RA than treatment with methotrexate alone or DMARDs combination. Similarly, Bae and Lee^16^ recently suggested that biosimilar- and originator-infliximab in combination with methotrexate is an effective intervention for active RA, with a low risk of adverse effects. Notably, the majority of our patients were on methotrexate as a choice of DMARD in combination with infliximab.

Previously, we have shared our experience with the use of biological therapy, specifically infliximab and rituximab, in the treatment of rheumatic diseases other than RA.^4^ In that study, we presented the efficacy of infliximab in treating rare rheumatic cases with minimal dramatic side effects. In the present study, we report comprehensive data on infliximab use for rheumatic diseases in a referral centre in the Arab region. Few other studies in the Arab region have assessed the efficacy of anti-TNF-α drugs including infliximab in treating several rheumatic diseases. Dewedar et al.^17^ from Egypt studied the adverse effects of TNF alfa inhibitors (infliximab, adalimumab and etanercept) in RA patients for 5 years in the south-west area of Saudi Arabia. These drugs appeared to be as safe as traditional DMARDs. Lutf and colleagues^18^ found in their cross-sectional study that the remission rate of RA in Qatar is better than that reported in other Gulf countries, which might be related to more use of anti-TNF in Qatar because it is supported by the government. Mansouri and colleagues^19^ from Morocco conducted a cross-sectional descriptive study on 117 AS patients in a tertiary referral rheumatology centre. Despite that, only 2.6% reported using anti-TNF-α therapy, those who used the drug did not report any TB complication. A recent collaboration by Dargham and colleagues,^20^ which assessed the epidemiology and treatment patterns of RA among a large cohort of Arab patients, found that only one-third of patients have ever used TNF-inhibitors. The incidence of TB in Lebanon is low (16 in 100,000),^21^ which explains the low incidence noted in our results (1 patient).

There are biases inherent to a cross-sectional analysis of any convenience sample. Recall bias resulting from underestimating the prevalence of co-morbidities and medication use is inevitable due to absence of information from the patients’ medical records.

The strength of the current data is that it is available from one centre where all rheumatologists use the same system and have standard protocols to use.

Despite such limitations, our study reflects clear data on infliximab use in rheumatic diseases in one of the referral centres in the Arab region. The significance of this study is that it investigates retrospective data since the start of use of infliximab for rheumatic diseases in the region taking into consideration the medical and socio-economic status of treated patients.

## CONCLUSION

In summary, this is a large retrospective medical chart review describing the use of the anti-TNF-α drug infliximab for treating chronic inflammatory diseases with its indications. Important observations include the low number of serious adverse events, the percentage of patients in remission, and the efficacy of cycling strategy. Future work should aim to study the efficacy of other biological drugs in chronic inflammatory disease to enhance patient and physician treatment preferences and maximize alternative options.
